# Experimental evidence supports the ability of spotted lanternfly to hitchhike on vehicle exteriors as a mechanism for anthropogenic dispersal

**DOI:** 10.1098/rsos.240493

**Published:** 2024-07-10

**Authors:** Johanna E. Elsensohn, Scott Wolford, Amy Tabb, Tracy Leskey

**Affiliations:** ^1^ USDA-ARS, Appalachian Fruit Research Station, Kearneysville, WV, USA

**Keywords:** non-random spread, human-assisted spread, spotted lanternfly, *Lycorma delicatula*, range expansion, population dispersal

## Abstract

Historically, anecdotal observations support the likelihood of human-assisted invasive insect dispersal to new environments. No previous studies have investigated the ability of insects to remain attached to moving vehicles; however, such information is critical for prioritizing research, mitigation activities and understanding anthropogenic effects on biotic communities. *Lycorma delicatula* (White), spotted lanternfly (SLF), an invasive insect whose range is currently expanding throughout the United States, is commonly observed in urban settings and near transportation hubs. We developed a novel method to test SLF’s ability to remain on vehicle surfaces including bonnet, nose wing, windscreen, wipers and scuttle panel using laminar wind flow from 0 to 100 ± 5 km h^−1^. We found all mobile life stages (nymphs and adults) could remain on the vehicle up to 100 km h^−1^. First instar nymphs and early season adults remained attached at significantly higher wind speeds than other stages. A brief acclimatization period prior to wind delivery increased attachment duration for all life stages except later season adults. The importance of outliers in the success of invasive species is well established. Given these results, any hitchhiking SLF could potentially establish incipient populations. This methodology will be beneficial for exploring human-assisted dispersal of other invasive arthropods.

## 1. Introduction

Accidental transport by humans is a key dispersal mechanism for invasive species [1–3]. Global trade is a main pathway for new species to enter a country, while domestic inter- and intra-regional travel can accelerate the spread of established populations [1,4,5]. Beyond natural dispersal mechanisms, the spread of invasive species, specifically invertebrates, happens in several ways, including their presence within plant-based food products, on plant material and by the adventitious travel (i.e. hitchhiking) of any insect life stage within or on modes of transportation or other non-living material. Invasive species threaten human health, the environment and food supply in addition to costing billions of dollars annually in lost revenue and mitigation efforts [6,7]. Because of their devastating impact, most countries have programmes to prevent the accidental release of novel species originating from overseas and to restrict their movement within country borders (e.g. [8]). Hitchhiking via means of conveyance (i.e. rail carriages, cargo ships or commercial and passenger vehicles) is thought to be an important, though not well understood, means of domestic dispersal for invasive species. Anecdotal reports proffer evidence for dispersal via hitchhiking for insects such as the emerald ash borer [9], while publications reviewing the progression of past insect invasions also note that hitchhiking probably assisted the dispersal of many invasive species [10–13].

Spotted lanternfly (SLF) (*Lycorma delicatula* (White)) is an invasive pest that was first detected in the United States in 2014 (Berks County, PA, USA), arriving by way of accidental human transport [14]. It is thought a shipment of stone imported from eastern Asia contained at least one viable SLF egg mass [14]. From 2014 to 2018, SLF population spread appeared isometric (electronic supplementary material, figure S1A). As the number of counties and states with SLF populations grew, so did the asymmetrical and disjunct nature of the population span. As of January 2024, over a dozen satellite populations of SLF were located distantly (at least two counties) from the main population block, as well as reported sightings of live SLF near Des Moines, Iowa ([15], electronic supplementary material, figure S1B). The current distribution shows biased spreading along major transportation corridors that include motorways and cargo rail lines (electronic supplementary material, figure S1B). SLFs have been observed on stationary railcars, lorries and passenger cars, and members of the public have posted images of SLF attached to moving cars of unknown velocity on social media platforms [16,17]. Diffusion and agent-based models also implicate human-assisted dispersal in this insect’s population spread in the United States [18–20]. The ability to attach, or hitchhike, on vehicles may be part of how SLF establish new satellite populations.

Insects are well known for their ability to climb vertical walls and walk upside down on virtually any surface, which is accomplished through multiple mechanisms (reviewed in [21]). There are two main categories of attachment type; the first are physical structures supported by mechanical force, such as tarsal claws or campaniform sensilla [22]. The second type involves other morphological features that have specific frictional properties to either maximize or minimize friction with another surface, such as dense hair patches, as seen in Diptera and Coleoptera, or smooth, flexible tarsal pads (e.g. arolia, euplantulae or pulvilli) present in Hymenoptera, Blattodea and some Hemiptera [21]. Like other Hemiptera, the presence of arolia on lanternfly tarsi significantly increases adhesion to smooth surfaces, while tarsal claws are advantageous on rough surfaces [23,24]. When tarsal claws are removed, an insect’s ability to remain attached to rough surfaces significantly decreases [25]. When disturbed, insects exhibit greater adhesion than under typical conditions [26]; in SLF, the surface area of sheared arolia was greater than those of perpendicular ‘resting’ arolia [23]. As SLF exhibit a cyclical climb–fall–climb behaviour pattern, SLF falling from trees may express different attachment behaviour than those at rest. Their climbing ability is positively associated with body and arolium size, an association shared among many climbing species of animals (see fig. 1 in [27]).

**Figure 1.**
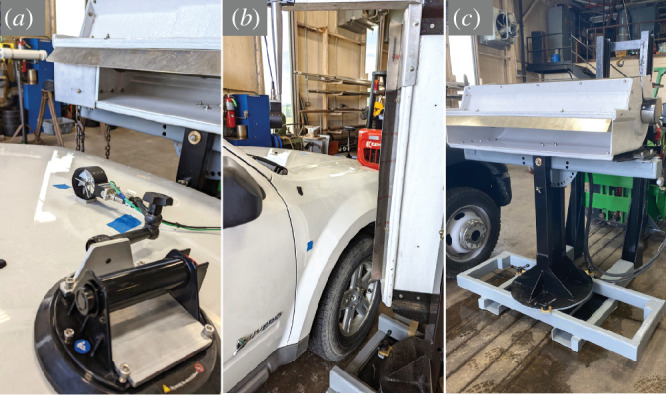
Experimental set-up of the laminar flow fan and vehicle placement. (*a*) Fan in horizontal position, situated just above and in line with the end of the bonnet. The anemometer was placed to the right of the tape mark, the insect starting position on the left. (*b*) The fan was rotated 90° into a vertical position when testing the offside nose wing. Insects began the assay just above the tape mark. (*c*) The fan was attached to a steel base; the power mover and the rest of the hydraulic system were located behind.

The practical applications of insect adhesion to other fields like aeronautics and engineering are far reaching, but this phenomenon has not been well explored with regard to invasion biology, barring one notable exception. Takano & Takasu [24] investigated the attachment ability of adult kudzu bugs, *Megacopta cribraria* (Fab.) to different surface textures. They placed a single bug on a glass microscope slide covered with nothing, cloth or aluminium, which were then exposed to vacuum forces up to 100 km h^−1^. Kudzu bugs remained on the cloth substrate at higher wind velocities than on glass or metal, suggesting insect adhesion is dependent on substrate texture, results supported using other species [24,28].

We present what may be the first methodological description of an empirical experiment to assess the ability of an insect to remain attached to vehicle surfaces when exposed to wind velocities up to 100 ± 5 km h^−1^. Since the population spread of SLF has been linked to human movement by anecdotal observations and statistical models [18–20], we asked the following questions: (i) Is hitchhiking on moving vehicles possible? (ii) How frequently could this travel be occurring? and (iii) Which of the mobile life stages remain attached to vehicles at high wind velocities? This work will serve as a model to better understand hitchhiking as a mechanism behind insect dispersal at short and long distances.

## 2. Methods

### 2.1. Insects

SLFs were sourced from individuals hatched in a greenhouse from egg masses collected in Mineral Co., WV, USA, in March 2022. SLF eggs were hatched in May and June by placing small pieces of bark covered by one or more egg masses in mesh cages (W32.5 × D32.5 × H77.0 cm, 680 µm aperture mesh, BugDorm-4S3074 Insect Rearing Cage, MegaView Science Co., Taiwan) with 30 cm tall potted tree of heaven (*Ailanthus altissima* (Mill.) Swingle) plants (see [29] for full methods). Only first and second instar nymphs were used from this rearing method in our assays. All other life stages were collected ad hoc from field populations in Mineral and Berkeley Cos., WV, USA. Field-collected insects were used in trials up to 2 days post-collection and were housed in cages with 30 cm tall tree of heaven saplings at 16 : 8 h (Light : Dark) 25°C and 60% relative humidity until use. The individuals used in assays were of known life stage but indeterminate age, although if an insect appeared to be freshly emerged into a new life stage (i.e. less than 24 h post-eclosion), they were not tested until the following day to allow full sclerotization and resumption of typical behaviour.

### 2.2. Laminar flow fan

The laminar flow fan was procured from an unused orchard sprayer (CX2000, Curtec Sprayers, Vero Beach, FL, USA). The fan assembly was removed from the sprayer frame, completely disassembled, cleaned, painted and a new fan rotor and bearings were installed. The opening of the fan exhaust was originally 1.18 m × 17.8 cm and rectangular in shape. This size opening did not produce the desired air flow output, so a removable baffle was added to one end, which restricted the outlet flow and in turn increased the velocity of the air expelled. The baffle constricted the fan exhaust area to 93.3 × 17.8 cm and could be placed on either side of the fan opening as needed.

A modular frame was constructed to allow various positions and rotations of the fan unit, so the air flow was directed over the vehicle bonnet in the horizontal position, or along the side of the vehicle in the vertical position. The frame was constructed with 50.8 × 76.2 mm and 50.8 × 104.8 mm steel tubing cut and welded together. The frame base was fastened to a turret that allowed the fan trusses to be rotated 360° on the vertical axis. In between the fan trusses was a cross beam with holes every 10° to allow fan rotation up to ± 90° (figure 1*b*).

### 2.3. Prime mover and hydraulics

The prime mover was an 18 horsepower (13.4 kW) Briggs and Stratton gasoline-fired, naturally aspirated engine operated at 3600 r.p.m. and delivered a hydraulic flow of 35.9 l min^−1^ (9.6 gal min^−1^) at 2700 pounds per square inch (psi) (186.15 Bar). This pressure was set through the relief valve; under static condition (fan rotor rotation stopped, engine idle) oil is diverted through the directional control valve back to the tank. Upon signal from the experimenter, the engine operator shifted the directional control valve, allowing oil to travel to the flow control valve. This valve was slowly and consistently turned open, allowing hydraulic fluid to pass into the hydraulic motor, causing the fan rotor to rotate and thus delivering air flow through the output of the fan exhaust. The maximum r.p.m. output of the blower rotation was 1850 r.p.m., thus the maximum wind speed output was 100 ± 5 km h^−1^ 60 cm from the housing exhaust. Wind speed output was measured using a 6.99 cm diameter wind vane anemometer (detailed in §2.4) and confirmed with a 2.54 cm diameter hand-held wind meter (Kestrel 1000, KestrelMeters, Boothwyn, PA, USA). Wind speeds were measured pre-trial at each testing location from 600 to 1850 r.p.m. in 100 r.p.m. increments. Anemometer output was remeasured before each life stage’s trials to ensure consistency over time.

A USDA federal vehicle was used in this experiment, a white 2011 Ford Escape Hybrid sport utility vehicle (SUV). The laminar flow fan was in the horizontal plane (0° position) for all vehicle locations tested except for the nose wing. In the horizontal plane, the fan was 1.24 m from the earth and the end of the exhaust outlet was even with the most forward part of the bonnet, with the baffle placed on the near side. In the vertical plane for the nose wing test, the fan was rotated 90° and the baffle was at the top of fan exhaust, with the bottom of the fan 0.56 m from the earth and 31 ± 0.5 cm diagonally from where the insect was placed. The insect starting position was 17.8 cm from the bottom of the nose wing panel (figure 1*b*).

### 2.4. Experimental details

**Figure 2.**
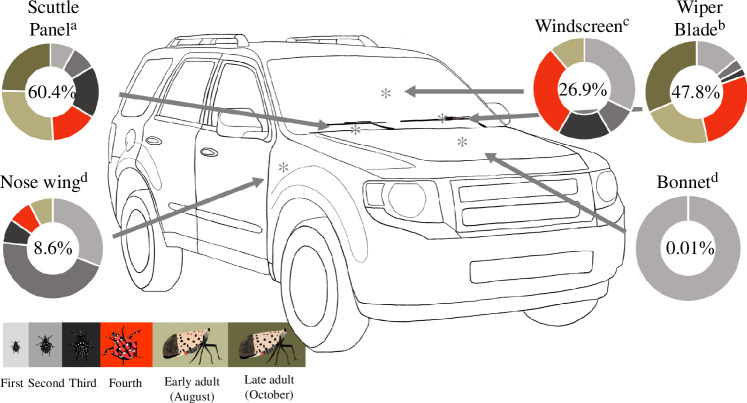
Successful adhesion by acclimatized insects until maximum wind speed was reached. For each vehicle location, the value within each circle represents the percentage of total insects that reached the maximum wind speed without detaching (*n* = 180 ± 2 insects per location except at nose wing (*n* = 151), due to the high number of non-responders, table 1). The colour and relative size of the circle segments represent the proportion of that percentage contributed by that life stage (see table 1 for numerical representation). The colour associated with each insect stage is defined by the background colour of the lower left panel. Only stages where at least one individual reached the maximum speed are displayed in the circle. Location on vehicle: *F*_4,868_ = 63.38, *p* < 0.001. Vehicle locations followed by the same letter are statistically equivalent at α = 0.05, Tukey test.

The following treatment locations and materials were used: (i) bonnet, white painted metal; (ii) front windscreen, clear auto glass; (iii) left wiper blade, black rubber with 3 cm black plastic cap, metal arm; (iv) scuttle panel, black plastic; and (v) offside nose wing, white painted metal (see figure 2 for approximate positions). The location where insects were placed was marked with 2 × 2 cm square of painter's tape; insects were placed 3–4 cm to the left of the tape mark and perpendicular to the wind machine at the start of each trial to reduce insect interaction with the tape. On the nose wing, the mark was set 4 cm below the insect starting position. The experiment took place in an open 8 m tall (two-storey) garage with the vehicle nose facing inward and the fan facing outward just in front of the vehicle grille. The floor-to-ceiling garage door was open to allow for free air flow. No artificial lighting was employed; indirect light from the door and windows was sufficient.

Wind speed was measured with a wind vane anemometer (AP275, 6.99 cm diameter probe head, Pacer Instruments, Keene, NH, USA), placed approximately 6 cm from the insect start location, in the same directional plane as the insect, adjacent to the tape mark. Pre-trial testing revealed no difference in wind speed measurement between the insect and anemometer locations. The anemometer was secured to the vehicle with a 20 cm diameter suction cup glass lifter (X002ZTT2HR, RSR Electronix Inc., Rahway, NJ, USA) modified with an arm to adjust the anemometer’s position and angle. Wind speed (km h^−1^) was recorded with a data logger (GL240, Graphtec America Inc, Irvine, CA, USA), which also recorded the motor rotation speed (r.p.m.). Data were recorded at 1 s intervals, began when the data logger was set to record and stopped recording when an attached trigger was depressed. Data recording commenced shortly before the insect was introduced to the vehicle.

Insects were used only once and transferred from the holding cage to the vehicle surface using a 29 × 95 mm cylindrical polypropylene vial (Flystuff.com, San Diego, CA, USA). Before the fan was initiated, insects were allowed an acclimatization period up to 5 s or until they initiated walking movement, whichever was sooner. At that point, the motor speed (r.p.m.) of the fan was manually increased until the test insect detached from the vehicle or the fan reached its upper speed threshold (1825 ± 25 r.p.m.), whichever was sooner. The acceleration pattern generated (electronic supplementary material, figure S2) resembled a Gompertz growth curve, a sigmoid function where increases in wind speed were slowest at the lower and upper bounds of allowable wind speeds. The mean time to reach the maximum wind velocity during the trials was 27.6 ± 0.44 s. The time for the wind speed to go from 0 to 100 km h^−1^ was approximately 15 s, calculated using the speed at peak acceleration (e.g. around the 7 s mark in electronic supplementary material, figure S2B). This type of acceleration mimics vehicle acceleration patterns in city and rural driving, but not for vehicles merging onto a motorway, which would require quicker acceleration to the top speed.

In this experiment, detachment was defined as the moment when all tarsi lost contact with the vehicle surface and the insect was moved elsewhere by the wind. Attachment was defined as remaining in contact with the vehicle surface in an upright position by one or more tarsi, and any movement from the starting location was initiated by the insect. At the moment of detachment, the stop trigger for the data logger was manually depressed, halting data collection and concluding the trial. We considered insects to be ‘non-responding’ if, after three attempts, the insect was unable to stay attached to the vehicle under any wind speed or it immediately jumped away. Trials for each testing location were repeated for the four nymphal instars and two time periods during the adult stage. Early season (pre-reproductive) adults were tested in August and early September, and later season (reproductive) adults were tested in late September and October [14]. For the adult trials, we recorded the sex and relative abdominal size by assessing the amount of yellow on the lateral part of the abdomen. Newly emerged adult SLF have a thin, almost completely black abdomen. The lateral sides of the abdomen may have a single narrow (less than 1 mm) yellow line, the line may be straight, slightly concave or absent during and just after sclerotization. Much of the first several weeks of the adult stage are spent feeding on plant phloem [30]; as a result, the abdomen increases in width and weight over time. The once narrow yellow lateral portion becomes larger and convex in appearance and correlates to overall weight [31]. For this trial, we considered an adult to be ‘small’ if the lateral yellow area was concave or flat and less than 2 mm wide. The insect was labelled ‘large’ if the lateral yellow area was greater than or equal to 2 mm wide and convex.

### 2.5. Testing no acclimatization time

An additional assay was conducted on all life stages to test attachment ability without an acclimatization period. We noticed in our exploratory assays (electronic supplementary material S1) that when exposed to low wind speeds, SLF would stop walking and either turn toward the wind or lower their centre of gravity toward the vehicle, also noted by Takano & Takasu [24]. In this assay, the insect was placed on the bonnet and exposed immediately to moving air, as opposed to starting the trial with no initial wind. We placed SLF on the bonnet and rather than starting at 0 km h^−1^, we used a starting wind speed that arrested the insect’s movement as soon as they were placed on the vehicle. Instead of being placed directly in the starting position, as in the previous assays, the insect was released from the holding vial approximately 5 cm above the testing surface. The trial began after the insect landed upright, which then proceeded in the same manner as before, increasing wind speed until detachment or reaching maximum km h^−1^. Similar to Kane *et al*. [32], we found ‘artificially releasing’ SLF resulted in a large percentage of insects failing to land upright upon impact. However, Kane *et al*. [32] also noted SLF would always land upright if they released themselves from an elevated surface. As an adjustment, we placed the vial directly upside down on the vehicle and tapped the insect onto the surface, ensuring the insect started in an upright position but not exposed to the wind pre-trial. The trial began when the vial was removed. Most third and fourth instar nymphs, and some adults required this alternative method to prevent landing upside down when released from the 5 cm height.

Early trials were conducted with the anemometer in a static position on the bonnet which did not allow us to measure the wind speed experienced by insects at each testing location. In later trials, we used a movable, dynamic anemometer version that allowed us to measure wind speed adjacent to the starting position. To join these two datasets, we used the linear relationship between the motor and wind speeds to generate a slope using the trial data collected at each location, allowing us to estimate a location-adjusted wind speed for earlier trial results. Motor speeds below 600 r.p.m. were excluded from these calculations, as the lower wind speeds were highly variable between trials. At least 250 data points were used to calculate the conversion equation; all equations had an *R*^2^ > 0.99 (electronic supplementary material, figure S3). Adult trial data were collected with the dynamic anemometer, while nymph wind speeds were estimated using the conversion calculation.

### 2.6. Statistical analysis

Data were analysed with R statistical software (v. 4.2.2) [33]. A two-way factorial (five locations (bonnet, scuttle, windscreen, nose wing, wipers) × six stages (first, second, third, fourth, early season adult, late season adult)) ANOVA model was fit using the *gls*() function of the *nlme* R package and specifying the *varIdent*() weight to indicate which of three magnitudes of observed within-treatment (among-replicates) variance each of the 30 combinations of location and stage exhibited. Variances of magnitude four times greater than other variances were considered to be different magnitudes. A log-likelihood ratio test indicated this heterogeneous variance model fit the data more accurately than the model assuming homogeneous within-treatment variances. Pairwise means comparisons among location means at each stage and among stage means at each location were conducted using the *emmeans* and *multcomp* packages, specifying the Tukey adjustment to maintain experiment-wise α < 0.05 [34,35].

To analyse the variables of sex and body size, we used Welch’s *F* test due to uneven sampling and variance among treatments. *Post hoc* pairwise mean comparisons using *α* < 0.05 were conducted to identify significant differences among treatment combinations. The ratio of treatment variances for acclimatization × stage was less than 3, so ANOVA could be used to provide appropriate statistical output. Pairwise means comparisons were conducted as before, specifying the Tukey–Kramer adjustment to maintain experiment-wise *α* < 0.05.

## 3. Results

### 3.1. Detachment wind speeds across life stages

**Figure 3.**
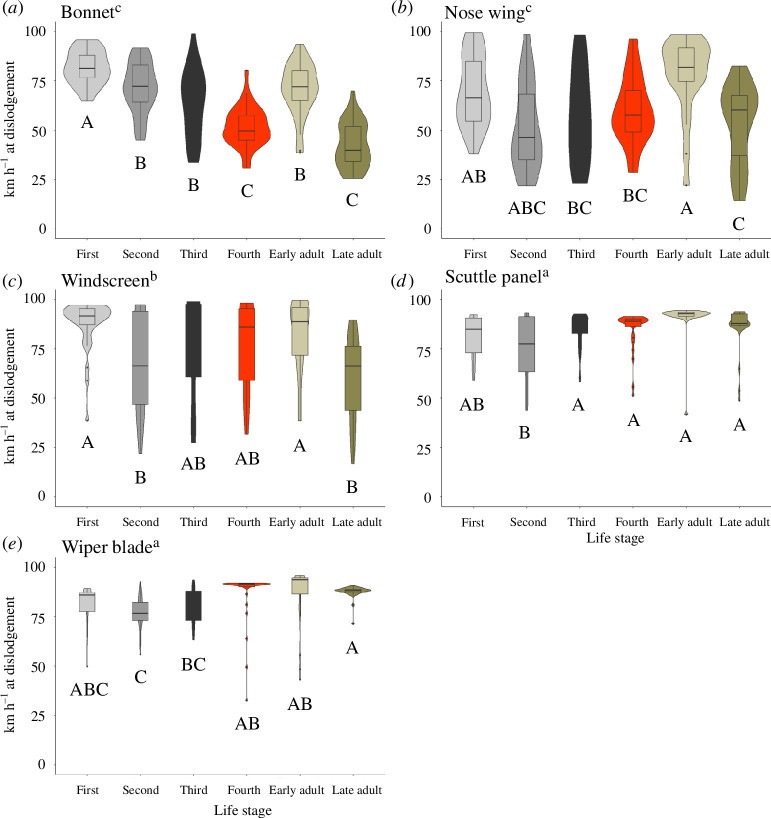
Mean detachment wind speeds at each vehicle location by insect stage. (*a*) Bonnet, (*b*) nose wing, (*c*) windscreen, (*d*) scuttle panel and (*e*) wiper blade. Location on vehicle: *F*_4,868_ = 46.24, *p* < 0.001. Letters following the vehicle location and under each life stage shows pairwise mean differences either between locations (lowercase) or among life stages within a given location (uppercase). Vehicle locations and life stages sharing the same letter and case are not statistically different at *α* = 0.05.

The wind speed at which acclimatized SLF detached from the car surface differed by both life stage of the insect and testing location (figure 3; location on vehicle: *F*_4,843_ = 113.24, *p* < 0.001; stage: *F*_5,843_ = 7085.29, *p* < 0.001). SLF on wiper blades or in the scuttle panel detached from the vehicle at the highest average wind speeds across life stages (83.8 ± 0.9 and 83.0 ± 0.8 km h^−1^ ± s.e.m., respectively), while individuals evaluated on the bonnet and nose wing detached at the lowest average wind speeds (64.0 ± 1.4 and 65.4 ± 1.9 km h^−1^, respectively). The interaction between location and life stage was significant; second instar SLF were least able to remain attached at high wind speeds in all locations evaluated except for the bonnet (location × stage: *F*_20,843_ = 12.74, *p* < 0.001). In contrast, first instar nymphs and early season adults remained on the vehicle at higher wind speeds on average across all locations (detachment speeds: 81.5 ± 1.1 and 82.6 ± 1.4 km h^−1^, respectively) and significantly outperformed all other life stages on the windscreen and nose wing tests. Later season adults yielded the greatest variation in performance by location; they detached at the lowest wind speeds on the bonnet, nose wing and windscreen, but exhibited sustained attachment among the highest wind speeds on the wipers and scuttle locations. Even so, the average wind speed at dislodgement for late season adult SLF across locations was the lowest among all life stages at 66.5 ± 1.9 km h^−1^.

### 3.2. Maximum wind speed

The percentage of total SLF that remained attached to the vehicle at the maximum wind speed was positively correlated with the mean detachment wind speed at each location. For example, 60% and 48% of all SLF tested were able to remain attached to the vehicle while experiencing maximum wind velocity in the scuttle panel and on the wiper blades, respectively (figure 2). These two locations also had significantly higher detachment wind speeds than at other locations (figure 3) and at least one SLF from each life stage remained attached until the maximum 100 ± 5 km h^−1^ wind speed was reached (table 1). Both life stage and location significantly affected adhesion to the vehicle at the highest wind speed (stage: *F*_5,843_ = 10.2, *p* < 0.001; location: *F*_4,843_ = 97.2, *p* < 0.001), and their interaction was also significant (*F*_20,843_ = 13.0, *p* < 0.001). Not all SLF life stages were able to remain attached until reaching the maximum wind speed at every location. However, individuals from all six stages reached the maximum speed at least once in two or more locations. No adults were able to reach the maximum speed on the windscreen. In contrast, adults represented about half the total insects still attached at the maximum wind speed in trials for the scuttle panel and wiper blades. The nose wing location had relatively few maximums attained, with 8.6% of tested insects able to withstand the highest winds without detaching, while the bonnet only had one insect, a first instar nymph, that stayed attached at the maximum wind speed (table 1).

**Table 1.** Successful attempts of vehicle attachment by SLF to reach maximum wind speed (reached max.) by insect stage and location. ‘total *N*’ is the number of insects tested for a given stage and location. ‘non-responding’ represents the number of insects tested unable to remain attached to the vehicle surface under any wind speed or immediately jumped away (see §2). Non-responders are not included in ‘total *N*’ and excluded from analysis. Life stages that share the same letter within a test location are not different statistically from each other. Tukey *α* = 0.05. Life stages with no letter designations were not significantly different from each other within a test location.

	spotted lanternfly life stage					
test location	first	second	third	fourth	early adult	late adult
**bonnet**						
reached max.	1	0	0	0	0	0
total *N*	31	30	28	30	30	29
non-responding	0	0	0	3	0	3
**nose wing**						
reached max.	4	6	1	1	1	0
total *N*	28	27	15	29	25	27
non-responding	10	14	14	1	6	5
**scuttle panel**						
reached max.	9^B^	9^B^	19^B^	17^BC^	29^A^	27^AC^
total *N*	30	31	33	28	30	30
non-responding	0	0	0	0	1	0
**windscreen**						
reached max.	17^A^	5^BC^	9^ABC^	12^AB^	6^BC^	0^C^
total *N*	31	31	29	30	31	30
non-responding	0	0	0	0	7	1
**wiper blade**						
reached max.	12^A^	3^A^	2^AB^	23^BC^	19^CD^	27^D^
total *N*	30	30	31	30	29	30
non-responding	1	0	0	0	2	0
**total/life stage**	**43**	**23**	**31**	**53**	**55**	**54**

### 3.3. Sex and abdomen size

**Figure 4.**
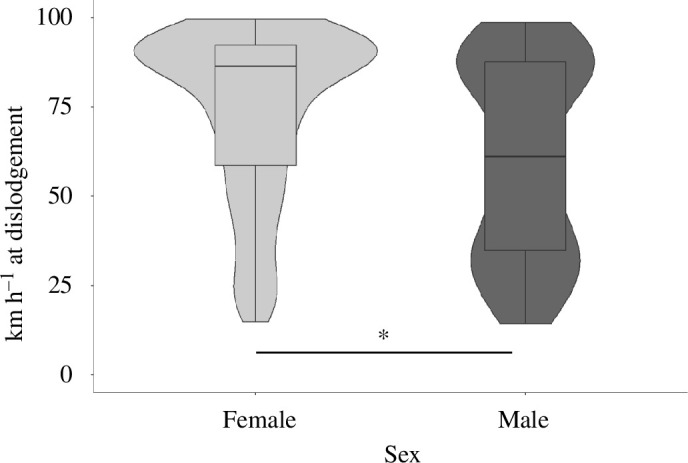
Effect of sex on insect attachment. Average wind speed at detachment of male and female SLF across all testing locations; *F*_1,139_ = 14.7, *p* < 0.001.

Sex had a significant impact on adult attachment, with females detaching at higher wind speeds compared with males (figure 4; females: 74.3 ± 1.5 km h^−1^, males: 61.5 ± 3.0 km h^−1^; *F*_1,139_ = 14.7, *p* < 0.001). We collected adult SLF from natural locations, so the sex ratio of tested insects was slightly biased to females as they were more abundant in our sampling area, with approximately one male for every two females.

**Figure 5.**
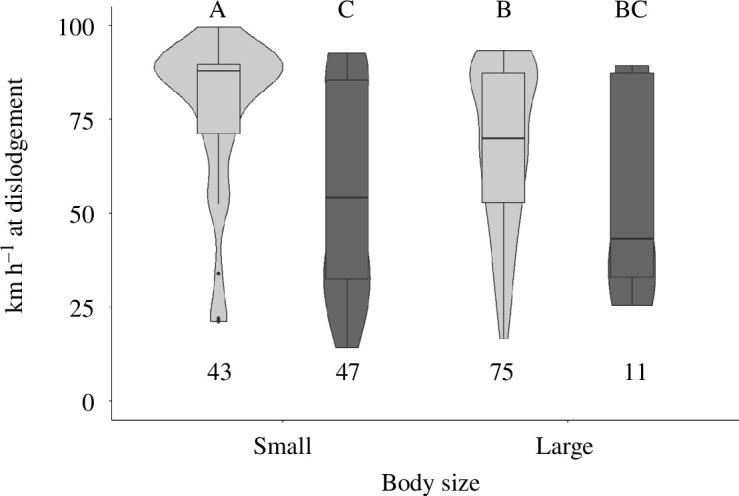
Effect of body size on adhesion ability of late season adult SLF. The number appearing below each bar represent the number of individuals (*n*) tested per category. Light grey bars represent female SLF, dark grey bars represent male SLF. *F*_3,41_ = 6.95, *p* < 0.001.

For later season adults, there was no effect of abdominal size on detachment wind speed (figure 5; *F*_1,174_ = 2.5, *p* = 0.11). During the early adult season, we captured fewer than five large adults, so we did not include early season data when analysing the size parameter. Sex and abdomen size significantly impacted detachment wind speed (*F*_3,41_ = 6.95, *p* < 0.001). Small-bodied female SLF detached at significantly higher average wind speeds than males and large females (figure 5).

### 3.4. Acclimatization time

**Figure 6.**
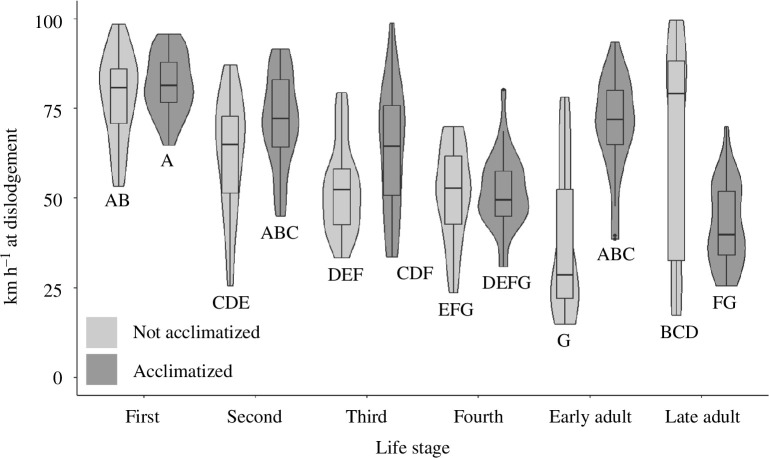
Effects of acclimatization versus no acclimatization. Acclimatization: *F*_1,348_ = 11.49, *p* < 0.001; life stage: *F*_5,348_ = 29.41, *p* < 0.001; acclimatization × life stage: *F*_5,348_ = 18.31, *p* < 0.001. Letters shared among insect life stages are not different statistically (Tukey test, *α* = 0.05).

Acclimatizing insects on the vehicle surface before exposure to wind significantly affected their detachment wind speed (*F*_1,348_ = 11.5, *p* < 0.001), especially for SLF adults, where acclimatization had opposing effects (acclimatization time * stage: *F*_5,348_ = 18.31, *p* < 0.001). Early season adults remained attached to the bonnet at higher wind speeds when acclimatized, while later season adults experienced better surface attachment when not acclimatized (figure 6). Acclimatization had no effect on nymph attachment, though across locations, acclimatized insects of a given stage detached from the vehicle at higher wind speeds than non-acclimatized insects of the same life stage.

## 4. Discussion

All life stages of SLF could remain attached to the vehicle against a maximum wind speed of 100 ± 5 km h^−1^ (approx. 62 miles h^−1^) at least once, while only first instar nymphs remained attached exposed to the highest wind speed at all five locations tested. Overall, approximately 30% of all SLF remained attached to the vehicle when exposed to maximum wind speeds, while the most favourable vehicle locations for insect adhesion (wiper blades and scuttle panel) had average dislodgement wind speeds of 83.5 km h^−1^. This capacity to withstand high wind speed suggests hitchhiking on vehicle surfaces may be a potentially significant mode of population dispersal for all mobile SLF life stages. How these insects can attach to various surface types under varying wind forces could be in part due to certain physical attributes.

The size and robusticity of the tarsal claws may have contributed to the relative adhesion success of fourth instar nymphs and adults placed on the wiper blades. The grip of tarsal claws has been shown to be a significant factor in insect adhesion of adults on uneven surfaces [24,36,37] while the size and age of the arolia influence attachment to smoother surfaces [23,27,38]. Frantsevich *et al*. [23] observed that SLF arolia adhesion to smooth surfaces appeared to decline with age and some older adults possessed blackened arolia versus the typical pink/light brown appearance, perhaps suggesting a lack of function. This ageing may the reason for the lower overall detachment wind speed for later season adults on the vehicle’s smooth surfaces. While this experiment was not designed to test the length of attachment against high wind speeds, our preliminary tests of adult female SLF on the wiper blade and subjected to 82 km h^−1^ winds were stopped after 8.5 min as the insects showed no signs of fatigue or distress (electronic supplementary material S1). Upon wind stoppage, these females immediately resumed typical movement. This resumption of behaviour was observed in the current experiment also; when wind speed dropped below the arresting speed, the SLF would begin walking perpendicular to, or away from, the wind source.

The variation in attachment ability among insect life stages may be driven in part by physical characteristics beyond tarsal morphology. Other attributes like body shape/size and leg length vary among nymphal instars and adults, and the combination of such traits seemed to impact surface attachment. For example, in response to wind exposure above the speed that would arrest their movement, all life stages of SLF would attempt to lower their centre of gravity closer to the vehicle surface. However, some life stages (adults, first instar nymphs) were more successful than others. Research on the contributions of various morphological traits to insect adhesion is needed to discern the influence of these potential mechanisms.

Where SLF were placed on the vehicle had a greater effect on adhesion ability than the life stage tested; nymphs and adults performed similarly across the locations tested. In general, second instars detached easily, having the lowest average detachment wind speeds, while first instar nymphs and early season adults detached at the highest wind speeds overall. Textured (wiper blades) or heterogeneously shaped (scuttle panel) surfaces resulted in insect attachment at higher wind speeds than on smooth surfaces like the metal panels and windscreen. The scuttle panel yielded the greatest percentage, 60%, of SLF able to stay attached up to the maximum wind speed tested, while the wiper blades allowed 48% of tested insects to withstand exposure to 100 ± 5 km h^−1^.

There was no difference in the average wind speed at detachment between SLF placed on a painted metal surface either vertically on the nose wing or horizontally on the bonnet. The nose wing location had the highest number of non-responding insects as all life stages of SLF had difficulty grasping onto or climbing the vertical surface. However, more individuals reached the maximum wind speed (13/151 total *N*) while only a single nymph reached the maximum on the bonnet (1/178 total *N*). The reason for this relatively poor performance may in part be due to the SLF’s physical limitations but also could be driven by the vehicle’s surface properties. For example, coatings that prevent insect residue is of significant interest for the transportation industry [39,40]. Residue from crushed insects after impact on vehicles and aeroplanes can increase drag and negatively influence fuel efficiency, affect a driver’s field of view through the windscreen, and be a cosmetic concern for many consumers, as acidic insect fluids aid in the erosion of a vehicle’s protective finish [28,40,41]. Typical wear and tear can alter the integrity of the vehicle surface, and thus insect attachment may be affected by damaged surfaces.

Our experiment is the first of its kind to directly test insect adhesion ability on a vehicle surface. We certainly recognize the limitations of this approach and suggest they be addressed by additional studies. One important limitation to note is the use of a single vehicle type in this experiment. While SUVs make up the majority (54% in 2022) of new vehicles produced and sold in the United States today, passenger lorries, minivans and sedans make up the remaining 46% [42]. On the one hand, vehicle profiles and attributes differ by manufacturer and model; any differences in vehicle aerodynamics could potentially impact attachment ability. On the other hand, vehicles share certain universal features, like the presence of a scuttle panel to vent air, or a wiper blade’s position and material composition. Our experiment sought to test the ability of an insect to hitchhike via vehicle exteriors, not to determine the influence of vehicle profiles or other attributes. The locations we tested are close to universally present on all vehicle types available today, which should enable comparison studies. Additional research is needed to investigate how vehicle model and profile affect SLF attachment ability at the same locations.

Furthermore, these trials were conducted without influence of rain, wind or direct sun, factors that may impact hitchhiking in natural settings. In our preliminary work, we conducted similar trials outside on days with and without rain. SLF adults on the windscreen were less able to remain attached under light rainfall conditions, as observed in other arthropods [43]. Without rain, the average wind speed SLF detached at was 58.7 ± 1.6 km h^−1^ (*n* = 52), while with rain the average speed was 39.0 ± 1.5 km h^−1^ (*n* = 30) (electronic supplementary material S1). Additional factors like cross winds and wind shear that can be experienced during typical driving conditions may also influence how long an insect can adhere.

Heat and temperature are related abiotic factors that could impact insect attachment and hitchhiking success too. Most insects are photosensitive, with their activity regulated by daily and annual swings in temperature and day length. SLF exhibit activity peaks in the afternoon hours [44] and most nymphs die when exposed to 35°C [45]. It follows that their attachment ability may also be affected by the temperature of the vehicle surface. Heat and sun exposure increase the temperature of absorbent surfaces like metal or glass, while running engines emit heat that can impact the temperature of adjacent surfaces. Insect behaviour can be affected by surface temperature, with small-bodied insects affected to a greater degree than larger insects [46–48]. Our experiment did not take the effect of temperature into consideration, as it was set in a covered garage and the vehicle engine was off. Our observed effect of acclimatization on attachment ability may have been influenced by habituation to vehicle surface temperature or texture. A more thorough understanding of these and other influencing factors will allow for a more specific approximation of insect dispersion by hitchhiking on vehicle exteriors.

Successful dispersal by hitchhiking insects requires more than just an ability to stay attached to a vehicle at motorway wind speeds. Post-detachment, the next step in founding a new population requires locating resources for survival and reproduction. In the United States, SLF’s current range expansion is heavily aided by the prior introduction and spread of their main host plant, tree of heaven, which began in the late eighteenth century [49,50]. Tree of heaven is closely linked to the spread and population size of SLF; this tree grows commonly in environmentally disturbed areas like edge habitats along roadsides and railways [49,51]. The accidental movement of SLF along transportation corridors where tree of heaven is abundant is likely to enhance spread of this species. Invasive and non-invasive species alike benefit from using pathways and corridors built by humans for their own travel. Bees [52], butterflies [53] and non-insect species [54] regularly use these open areas for dispersal, often more so than other land types. Dispersal along open corridors like rail lines, motorways and rights-of-way may require less energy to navigate, and thus may be used for movement over non-open habitats. The ease of corridor movement along with readily accessible host species for SLF [55] may underpin the occurrence of isolated SLF satellite populations.

In non-forest settings, adult SLF have been observed gliding from one tree to another after launching off tall objects, with longer distance flights reaching up to 40 m [56]. Mass dispersal flights also have been observed [30], but distances flown by these adults were not measured. Thus, while flight dispersal by SLF is likely over short distances, there may be periods of time where longer distances are travelled. This could lead to increased spread on a local scale or enhanced hitchhiking opportunities as these dispersers have been observed swarming and landing in parking lots and on other human-made objects [30].

While our results may overestimate the likelihood of long-distance travel by SLF on vehicles due to the favourable experimental conditions discussed above, they also highlight the potential for and importance of outliers in invasive species spread. Only one gravid female is needed to begin a population of a species in a new area, and SLF females can deposit multiple egg masses and lay hundreds of eggs in their lifetime [29]. While small initial populations often fail and go extinct from various demographic and environmental stochasticity, many successful invasive species began the establishment phase with low founding genetic diversity [57–60]. Together, this suggests that each of the dozens of SLF satellite populations that appeared in 2023 and in years prior could have resulted from a single or multiple introduction event due to the hitchhiking mechanism presented in this study and factors discussed above. In 2023 alone, five new satellite populations were identified.

Adult SLF probably have the highest likelihood of establishing new satellite populations through hitchhiking. Although not well understood, a large percentage of mating appears to take place four to six weeks post-eclosion to the adult stage [14,61]. In our study, adult females generally showed greater attachment success than males independent of age (i.e. season) or abdomen size. While the current experimental design limits our ability to extrapolate these data to performance under real-world conditions, the strong attachment capability observed for adult females specifically indicates a higher likelihood of dispersal by means of vehicle hitchhiking. In other words, the life stage most capable of establishing new population sites, through the production of viable offspring, also had the most success in withstanding exposure to high winds.

Our results confirm anecdotal evidence that SLF can hitchhike on vehicles for an indeterminate time and could help improve models forecasting potential spread along corridors. The data can also complement technologies like eDNA (environmental DNA), which can detect SLF presence even at low population densities, when traps and human observers may not detect their presence [62]. If resulting models predict high likelihood of spread along a particular corridor, eDNA could be used to assess and confirm presence. Allen *et al*. [62] collected SLF eDNA, probably honeydew residue, by spraying water on selected host plants and testing the recollected liquid. This technique can be used on any surface onto which SLF might excrete honeydew or oviposit egg masses. This will allow for quicker identification of new populations, as eDNA shows improved SLF detection rates over visual surveys alone [62].

Similarly for invasive species risk management, even adverse events that have a low probability will happen given enough opportunity. Inadvertent human-assisted transport of this species may explain the rapid spread of SLF to new locations. A greater focus on reducing hitchhiking as a significant dispersal mode may enhance current mitigation efforts. Ultimately, as SLF continues to spread and threaten economically important crops such as wine grapes [61,63], it will be critical to understand the mechanisms underlying this insect’s spread to and establishment in new areas to ensure affected producers can be prepared to manage this invasive pest.

## Data Availability

The primary dataset is available at [64]. Supplementary material is available online [65].
